# Comprehensive Analysis of Lung Adenocarcinoma and Brain Metastasis through Integrated Single-Cell Transcriptomics

**DOI:** 10.3390/ijms25073779

**Published:** 2024-03-28

**Authors:** Vanessa G. P. Souza, Nikita Telkar, Wan L. Lam, Patricia P. Reis

**Affiliations:** 1 Molecular Oncology Laboratory, Experimental Research Unit, Faculty of Medicine, São Paulo State University (UNESP), Botucatu 18618-687, SP, Brazil; 2 British Columbia Cancer Research Institute, Vancouver, BC V5Z 1L3, Canada; 3 British Columbia Children’s Hospital Research Institute, Vancouver, BC V5Z 4H4, Canada; 4 Department of Surgery and Orthopedics, Faculty of Medicine, São Paulo State University (UNESP), Botucatu 18618-687, SP, Brazil

**Keywords:** lung cancer, NSCLC, tumor microenvironment, brain metastasis

## Abstract

Lung adenocarcinoma (LUAD) is a highly prevalent and lethal form of lung cancer, comprising approximately half of all cases. It is often diagnosed at advanced stages with brain metastasis (BM), resulting in high mortality rates. Current BM management involves complex interventions and conventional therapies that offer limited survival benefits with neurotoxic side effects. The tumor microenvironment (TME) is a complex system where cancer cells interact with various elements, significantly influencing tumor behavior. Immunotherapies, particularly immune checkpoint inhibitors, target the TME for cancer treatment. Despite their effectiveness, it is crucial to understand metastatic lung cancer and the specific characteristics of the TME, including cell–cell communication mechanisms, to refine treatments. Herein, we investigated the tumor microenvironment of brain metastasis from lung adenocarcinoma (LUAD-BM) and primary tumors across various stages (I, II, III, and IV) using single-cell RNA sequencing (scRNA-seq) from publicly available datasets. Our analysis included exploring the immune and non-immune cell composition and the expression profiles and functions of cell type-specific genes, and investigating the interactions between different cells within the TME. Our results showed that T cells constitute the majority of immune cells present in primary tumors, whereas microglia represent the most dominant immune cell type in BM. Interestingly, microglia exhibit a significant increase in the COX pathway. Moreover, we have shown that microglia primarily interact with oligodendrocytes and endothelial cells. One significant interaction was identified between DLL4 and NOTCH4, which demonstrated a relevant association between endothelial cells and microglia and between microglia and oligodendrocytes. Finally, we observed that several genes within the HLA complex are suppressed in BM tissue. Our study reveals the complex molecular and cellular dynamics of BM-LUAD, providing a path for improved patient outcomes with personalized treatments and immunotherapies.

## 1. Introduction

Lung cancer is the leading cause of cancer-related deaths [1]. Lung adenocarcinoma (LUAD) is the most common histological subtype of lung cancer, accounting for roughly 40–50% of all lung cancer cases [2,3]. LUAD is commonly detected at advanced stages with regional and/or distant metastasis that can affect the brain, leading to the currently observed high mortality rates. Compared to other types of lung cancer, LUAD has a higher tendency to metastasize to the brain [4,5]. The process of brain metastasis (BM) involves several steps, including cancer cells migrating to the brain, establishing a metastatic focus, and interacting with the brain’s microenvironment [6]. Managing BM is one of the most difficult clinical challenges, requiring multidisciplinary approaches, mainly comprising local interventions such as surgery, radiotherapy, and palliative care, including treatment with corticosteroids [6]. However, conventional therapies offer only marginal survival benefits and are often associated with high morbidity rates due to neurotoxic effects, which may lead to cognitive impairment and other neurological complications [7,8,9].

The tumor microenvironment (TME) is a complex ecosystem where cancer cells interact with immune cells, stromal cells, blood vessels, and the extracellular matrix and where these interactions can significantly impact tumor behavior and cancer progression [10]. In the context of immunotherapies, targeting the TME is an attractive strategy for treating cancer [11]; for example, immune checkpoint inhibitors (ICIs) are antibody-based therapies targeting immune cells in the TME [12]. ICIs were approved as early as 2011 to treat unresectable advanced melanoma after conventional therapy [13], and, as of September 2022, the US Food and Drug Administration (FDA) has approved nine drugs targeting four immune checkpoints, including cytotoxic T-lymphocyte-associated protein-4 (CTLA-4), programmed cell death-1 (PD-1), programmed death ligand-1 (PD-L1), and lymphocyte activation gene-3 (LAG-3).

Over the past decade, immunotherapy with ICIs has seen a significant breakthrough in advanced lung cancer treatment, offering patients substantial improvements in terms of survival and quality of life [14]. In the context of BM treatment, emerging data suggest that ICIs exhibit promising activity and safety in non-small cell lung cancer (NSCLC) patients with BM [15]. However, more studies are needed to understand the molecular features that characterize metastatic lung cancer and the complex microenvironment supporting its progression. A comprehensive understanding of these factors is crucial for refining treatment strategies and improving patient outcomes. Moreover, understanding the heterogeneity of the TME allows for the identification of potential biomarkers that may predict responses or resistance to immunotherapies. Tailoring treatment strategies based on the unique characteristics of the TME may improve patient outcomes and expand the success of immunotherapies in BM from LUAD (BM-LUAD).

Studies utilizing single-cell transcriptomic sequencing (scRNA-Seq) have successfully unveiled pivotal molecular insights into individual metastatic cells in lung cancer and BM, where these investigations have provided valuable findings concerning the molecular and cellular reprogramming of metastatic lung adenocarcinoma, the tumor heterogeneity in lung cancer, and the diversity present in the tumor microenvironment of BM [16,17,18,19]. Nonetheless, one of the main challenges in studying molecular changes associated with BM is the limited number of samples for analysis, as BM is usually not resectable, leaving only the primary tumors accessible.

**Figure 1:**
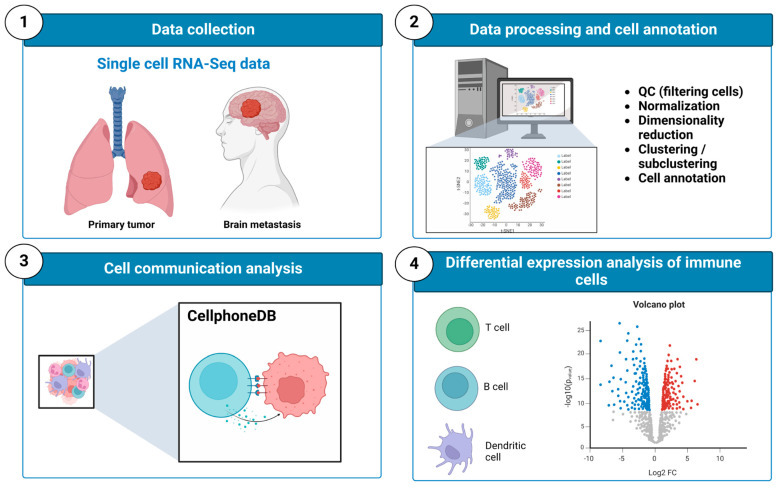
Study design: three independent datasets were collected from GEO (see Table 1) and subsequently underwent pre-processing, quality control, normalization, and clustering using the Seurat package. Dataset integration was conducted using an anchor-based approach, with annotations derived from the CellMarker2.0 database. Subsequent cell communication analysis unveiled interactions between cells. Following this, differentially expressed genes between immune cell types were identified using the FindMarkers function from the Seurat package.

In order to address this challenge, we integrated multiple scRNA-seq datasets derived from BM-LUAD and primary tumors across various stages (I, II, III, and IV), outlined in Figure 1. Our primary focus was the immune cell composition and the expression profiles and functions of cell-type-specific genes. Additionally, we explored cellular interactions within the TME by obtaining cell–cell communication mechanisms by utilizing a database of ligands and receptors. Overall, our study provides additional insights for further research on the TME-immune ecosystem and immunotherapy for BM-LUAD.

## 2. Results

### 2.1. Processing for Single-Cell RNA Sequencing Data

**Figure 2:**
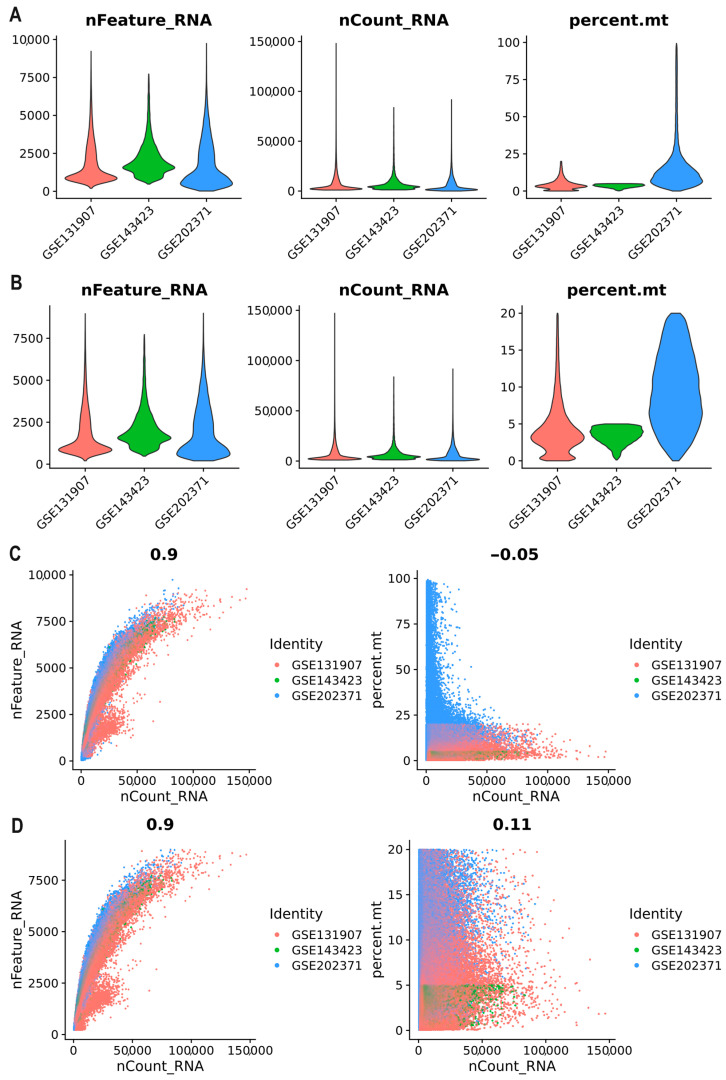
Quality of the final dataset. Violin plots depict QC metrics of cells in the final dataset, illustrating the number of unique genes detected in each cell (nFeature_RNA), the total number of molecules detected within a cell (nCount_RNA), and mitochondrial gene expression (percent_mt), categorized by study. (**A**) Violin plots before quality control. (**B**) Violon plots after quality control. Additionally, FeatureScatter plots visualize feature–feature relationships before (**C**) and after (**D**) quality control analysis.

We obtained the expression data from three scRNA-Seq datasets available in the Gene Expression Omnibus (GEO) database. These datasets included 23 samples from BM and 15 samples from primary tumors, covering stages I, II, III, and IV of disease (Table 1). The total number of cells examined was 148,905, and from these, we evaluated the number of unique genes detected in each cell to identify low-quality cells, empty droplets, or cell doublets/multiplets (nFeature_RNA) (Figure 2A). Additionally, we assessed the total number of molecules detected within a cell (nCount_RNA) and mitochondrial gene expression (percent_mt) (Figure 2A). Elevated expression levels of mitochondrial genes could indicate poor sample quality, suggesting a high occurrence of cell apoptosis or lysis and low cell activity.

A total of 136,946 cells (GSE131907, *n* = 86,274; GSE143423, *n* = 12,196; and GSE202371, *n* = 38,476) were identified after quality control. Only cells meeting the quality criteria of nFeature_RNA > 200 and <9000, and percent.mt < 20 were retained for downstream analysis (Figure 2B). We employed a FeatureScatter plot to visualize feature–feature relationships before quality control (Figure 2C) and after quality control (Figure 2D). Figure 2D displays a positive correlation of 0.90 between the sequencing depth and the number of detected genes. This positive correlation is desirable, as it implies that a higher sequencing depth results in a more comprehensive and accurate representation of the cellular transcriptome. After conducting quality control filtering, we detected a positive correlation of 0.11 between the sequencing depth and mitochondrial gene content. This means that as the sequencing depth increases, the mitochondrial gene content also tends to increase. However, the correlation is weak, which suggests that other factors may also influence the mitochondrial gene content.

### 2.2. Principal Component Analysis and Batch Effect Correction

**Figure 3:**
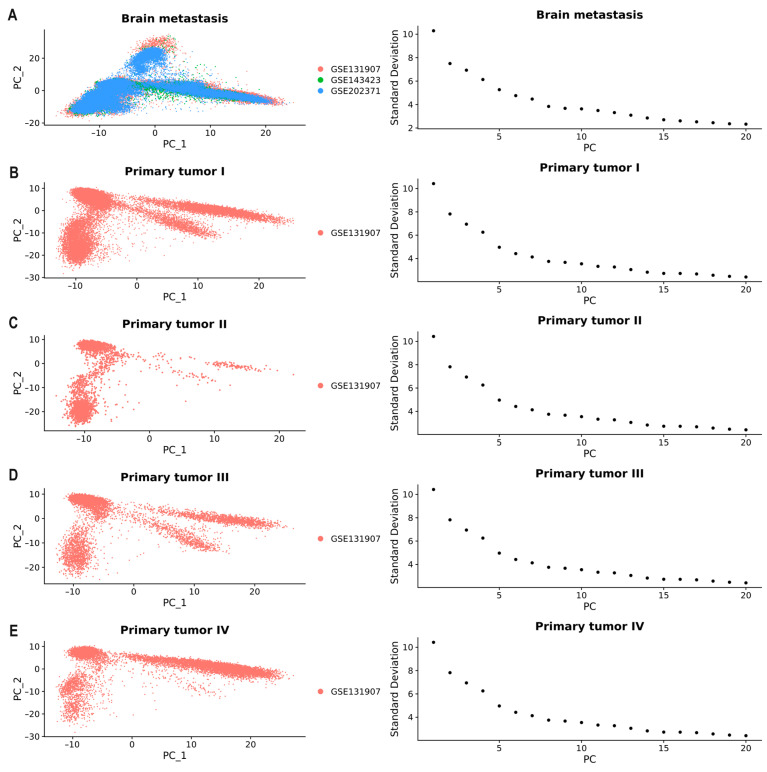
DimPlots and elbow plots display PCA results for (**A**) Brain metastasis. (**B**) Primary tumor stage I. (**C**) Primary tumor stage II. (**D**) Primary tumor stage III. (**E**) Primary tumor stage IV.

After quality control analysis, datasets were integrated using identified anchors, and downstream analysis was conducted. Principal component analysis (PCA) was utilized to select a set of linearly independent variables known as principal components (PCs). The Elbowplot function helped determine the number of principal components, showcasing the contribution of each PC ranked according to the percentage of variance. The elbow plot revealed an ‘inflection point’ around PC15, signifying that the first fifteen principal components captured most of the true signals. Consequently, we selected these 15 principal components for subsequent analysis (*p* < 0.05). PCA was then performed on the expression data for dimensionality reduction analysis (Figure 3). The integration of the three datasets of BM samples after normalization and batch effect correction is presented in Figure 3A.

### 2.3. Primary Tumors and Brain Metastases Exhibit Different Immune and Stromal Infiltration Patterns

**Figure 4:**
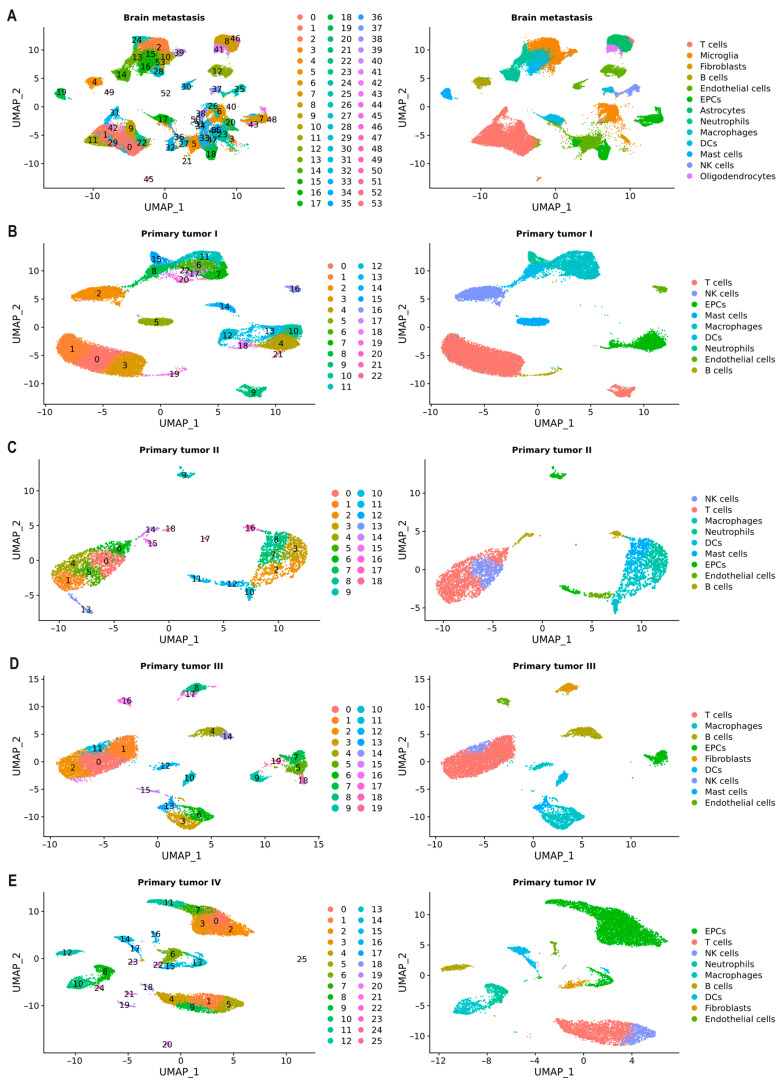
UMAP plots showing the main cell types annotated by known gene markers. (**A**) Brain metastasis. (**B**) Primary tumor stage I. (**C**) Primary tumor stage II. (**D**) Primary tumor stage III. (**E**) Primary tumor stage IV. DCs: dendritic cells. EPCs: epithelial cells. NK cells: natural killer cells.

After data processing and quality control, we successfully cataloged 136,946 cells, each assigned to distinct cell types through annotation based on expressed marker genes. Our classification comprised five non-immune cell types, including endothelial cells, fibroblasts, epithelial cells (EPCs), astrocytes, and oligodendrocytes (the latter two only found in BM), alongside eight immune cell types: macrophages, dendritic cells (DCs), neutrophils, mast cells, T cells, natural killer (NK) cells, B cells, and microglia (present exclusively in BM) (Figure 4).

We applied the Uniform Manifold Approximation and Projection (UMAP) method for sub-clustering and classified the cells from BM samples into 54 separate clusters (Figure 4A), primary tumor stage I (TI) into 23 separate clusters (Figure 4B), primary tumor stage II (TII) into 19 clusters (Figure 4C), primary tumor stage III (TIII) into 20 clusters (Figure 4D), and, finally, primary tumor stage IV (TIV) into 26 clusters (Figure 4E). Then, we annotated the clusters as described in the methodology.

In BM samples, we identified immune cells, including macrophages (*n* = 2734), DCs (*n* = 4447), neutrophils (*n* = 5910), mast cells (*n* = 732), T cells (*n* = 13,818), NK cells (*n* = 1861), B cells (*n* = 873), and microglia (*n* = 14,813); and non-immune cells, which included endothelial cells (*n* = 7267), fibroblasts (*n* = 517), EPCs (*n* = 8698), astrocytes (*n* = 4211), and oligodendrocytes (*n* = 911) (Figure 4A). Functional enrichment analysis suggested that the COX reactions pathway is significantly upregulated while the ATP-sensitive potassium channels pathway is downregulated in macrophages, microglia, DCs, mast cells, and EPCs (Supplementary Figure S1). Furthermore, all of these cells exhibit the upregulation of gene and protein expression by JAK-STAT signaling via the Interleukin-12 stimulation pathway (Supplementary Figure S2). Table 2 shows the number of cells per tumor stage in primary tumor samples. It is important to note that TIV showed no presence of mast cells and in TIII there were no neutrophils. Also, the fibroblasts were too few to detect in stages I and II (Figure 4B–E).

**Figure 5:**
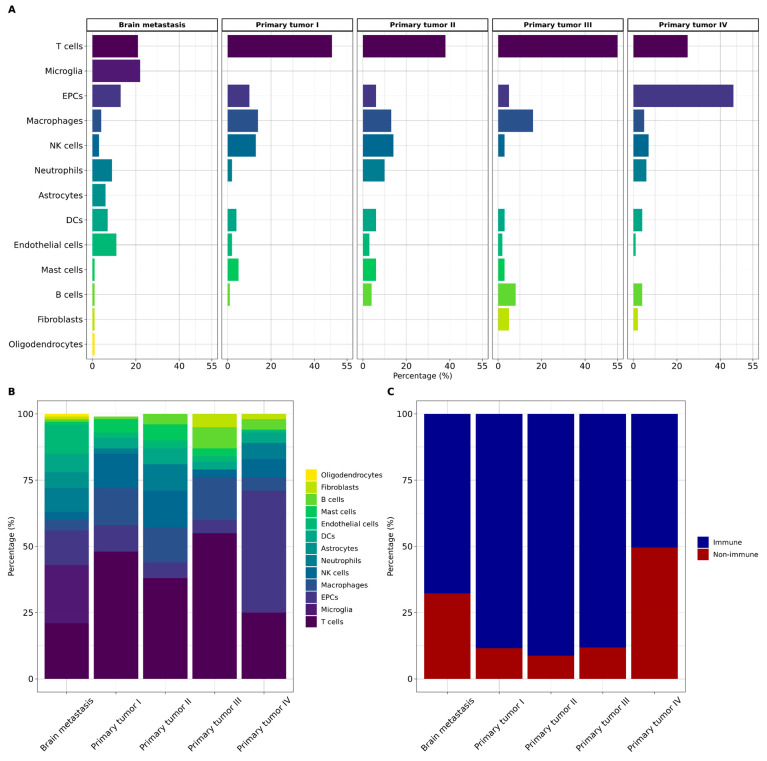
(**A**,**B**) The bar plot displays a comparison of the proportions of the main cell types between brain metastasis and primary tumors across four stages (I, II, III, and IV). (**C**) The bar plot shows the composition of immune and non-immune cells in both brain metastasis and primary tumors. DCs: dendritic cells. EPCs: epithelial cells. NK cells: natural killer cells.

Subsequently, we quantified the proportion of each cell type in both primary tumors and BM (Figure 5A,B). Notably, immune cells exhibited higher proportions in both primary tumors and BM (Figure 5C), constituting 87% for TI, 91% for TII, 88% for TIII, 51% for TIV, and 68% for BM. T cells emerged as the predominant immune cell type across all primary tumors, representing 48%, 38%, 55%, and 25% for stages I, II, III, and IV, respectively. Meanwhile, microglia (22%) stood out as the primary immune cell type in BM.

Moreover, TI is marked by a 14% presence of macrophages, accompanied by significant contributions from NK cells, EPCs, and DCs. In TII, NK cells show prominence at 14%, while macrophages, neutrophils, DCs, and mast cells collectively constitute 40%. TIII is characterized by 16% macrophages and 8% B cells, with EPCs, fibroblasts, DCs, and NK cells contributing a combined 20%. TIV is distinguished by featuring EPCs as the major component, at 46%, alongside NK cells, neutrophils, and macrophages, constituting 19%. B cells, DCs, fibroblasts, and endothelial cells collectively account for 13% (Supplementary Table S1).

Functional enrichment analysis indicates that most primary tumors downregulated the FGFR1c and Klotho ligand binding and activation pathway (Supplementary Figures S3–S6).

### 2.4. Complex Intercellular Communication Networks in Brain Metastasis

**Figure 6:**
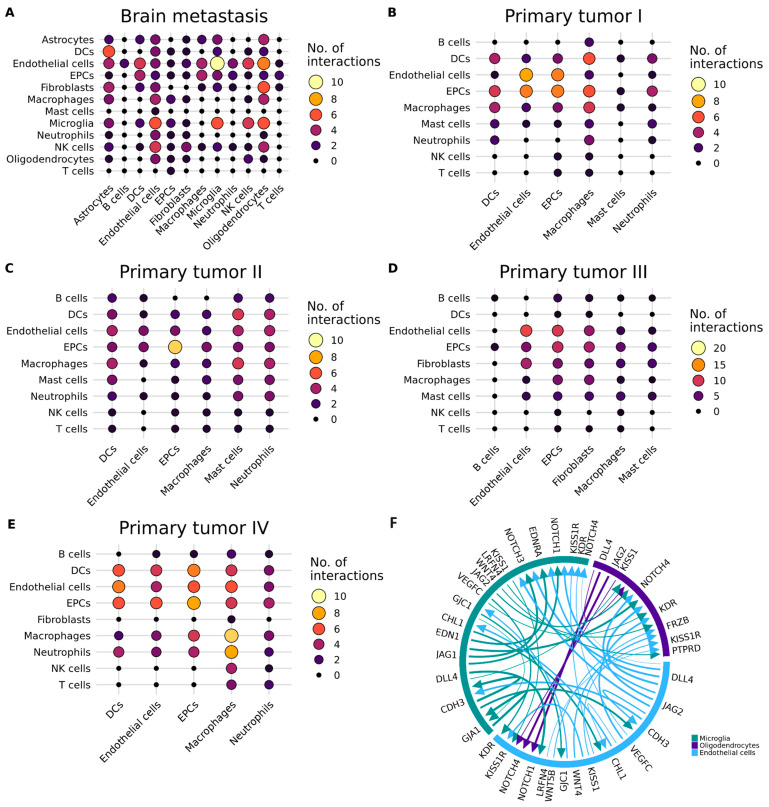
Inferred cell–cell signaling through ligand–receptor interaction analysis in CellPhoneDB. (**A**–**E**) Number of interactions between the cell types. Point size and colors represent the number of ligand–receptor interactions (*p* < 0.05). Node size represents the number of interactions. (**F**) Selected ligand–receptor interactions between microglia, oligodendrocytes, and endothelial cells in brain metastasis samples. The colors represent the cell types and the arrows represent the interaction between ligands and receptors. DCs: dendritic cells. EPCs: epithelial cells. NK cells: natural killer cells. No.: number.

Next, we identified ligand–receptor pairs and molecular interactions among the major cell types (Figure 6A–E). Broadcast ligands, for which cognate receptors were detected, demonstrated extensive communication between immune and non-immune cells, underscoring their crucial roles in the communication between immune and non-immune cells within the TME in the development and progression of lung cancer.

Notably, BM exhibited a higher number of significant (*p* < 0.05) potential interactions compared to primary tumors (Supplementary Tables S2–S6). Remarkably, in BM samples, endothelial cells exhibited a higher number of interactions with both microglia and oligodendrocytes (10 and 8, respectively) (Figure 6A). Additionally, by using this integrative approach, we were able to identify that microglia primarily interact with oligodendrocytes and NK cells.

Among the most remarkable findings, a pivotal interaction was identified between *DLL4* and *NOTCH4*, demonstrating a significant (*p* < 0.05) association between endothelial cells and microglia and between microglia and oligodendrocytes. Another noteworthy interaction was found between *VEGFC* and *KDR*, revealing a significant association between microglia and oligodendrocytes. Additionally, the interaction between *LRFN4* and *PTPRD* showed a significant association between endothelial cells and oligodendrocytes (Figure 6F).

Additional notable findings included *KISS1*-*KISS1R* interactions, associated both between microglia and endothelial cells and between oligodendrocytes and endothelial cells. Similarly, the interaction between *WNT4* and *FRZB* demonstrated a significant association between microglia and oligodendrocytes (Figure 6F). WNT4 and FRZB are two proteins involved in the Wnt signaling pathway, which regulates key cellular events during the development of the brain and is involved in the genesis of glioblastoma [20].

Additionally, we observed distinct cell–interaction profiles in primary tumors, with fewer interactions between immune and non-immune cells in stages I, II, and III (as shown in Figure 6B–D). However, from Stage IV onwards, we noted an increase in interactions between the immune and non-immune cells, with macrophages exhibiting a higher number of interactions, mainly with neutrophils, DCs, endothelial cells, and EPCs. It is noteworthy that previous studies have demonstrated the involvement of macrophages in tumor growth, migration, and metastasis [21], which aligns with the results that we obtained.

### 2.5. Immune Cells Reveal Potential Roles of the Human Leukocyte Antigen Complex (HLA) in Brain Metastasis Progression

Following the comprehensive characterization of cell types within each sample group, we proceeded to investigate the distinct expression patterns of genes, known as differentially expressed genes (DEGs), within specific cell types across different stages of primary tumors (I, II, III, and IV) and BM. We specifically focused on the differential expression profiles of various immune cell populations present in both primary tumors and BM, such as T cells, macrophages, NK cells, neutrophils, DCs, mast cells, and B cells. The aim was to identify and elucidate differentially expressed genes for each specific cell type, thereby shedding light on the molecular intricacies underlying the immune landscape across different stages and tissue environments.

**Figure 7:**
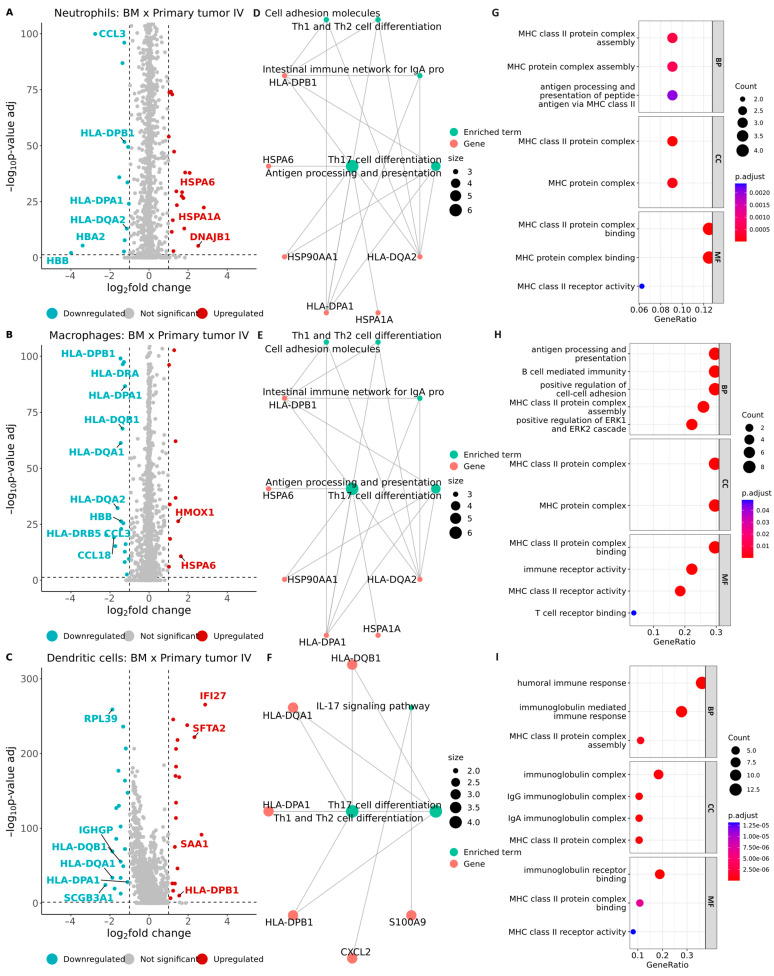
Differential expression of genes in specific cell types between brain metastasis and primary tumors at stage IV. Volcano plots show −log10 adjusted *p*-value on the *y*-axis versus log2 fold change on the *x*-axis. (**A**) Neutrophils. (**B**) Macrophages. (**C**) Dendritic cells (DCs). The labels indicate genes that are part of the human leukocyte antigen (HLA) complex. Additionally, representative genes with highly significant fold changes are shown in the volcano plot. See Supplementary Tables S8, S10, and S11 for full lists of significantly changed genes in macrophages, neutrophils, and DCs, respectively. (**D**–**F**) Enrichment analysis interaction network from the Kyoto Encyclopedia of Genes and Genomes (KEGG), for neutrophils, macrophages, and DCs, respectively. The node size represents the number of genes according to each KEGG category, and the color of the nodes represents the enriched term (green) and gene (red), as shown by the legend. (**G**–**I**) Enrichment dot plot of the term “Genetic Ontology” (GO). The graph displays the enriched ontologies associated with the genes presented in the volcano plot. Each of the instance terms (BP = biological process, MF = molecular function, and CC = cellular component) is represented (*p* < 0.05). The *X*-axis presents the number of genes that enrich the ontology term, and the point size is proportional to this number.

Distinct profiles of gene expression emerged across different comparisons (refer to Supplementary Tables S7–S13). Notably, we observed the downregulation of several genes within the human leukocyte antigen (HLA) complex, including *HLA-DPB1* (log2FC = −1.25), *HLA-DPA1* (log2FC = −1.03), and *HLA-DQA2* (log2FC = −1.13) in neutrophils from BM when compared to neutrophils from primary tumors at stage IV (Figure 7A). Similarly, in macrophages from BM, we observed the downregulation of several genes within the HLA complex (*HLA-DPB1*, *HLA-DRA*, *HLA-DPA1*, *HLA-DQB1*, *HLA-DQA1*, *HLA-DQA2*, and *HLA-DRB5*) when compared to macrophages from primary tumors at stage IV (Figure 7B). These genes enriched pathways such as Th1 and Th2 cell differentiation and Th17 cell differentiation (Figure 7D–F). Furthermore, subsequent Gene Ontology (GO) enrichment analysis demonstrated that these genes were associated with processes such as MHC class II protein complex assembly, peptide antigen assembly with MHC class II protein complex, and antigen processing and presentation (Figure 7G–I). The results were similar for DCs from BM when compared to DCs from primary tumor stage IV (Figure 7C,F,I). The results of the GO enrichment analysis and Kyoto Encyclopedia of Genes and Genomes (KEGG) can be found in Supplementary Tables S14 and S15, respectively.

Additionally, T cells, among the most abundant immune cells in all samples, exhibited significant gene dysregulation in the BM compared to primary tumors. Specifically, genes such as *PLP1*, *LUM*, and *IGFBP7* showed substantial alterations (|log2FC| > 2) in T cells from BM when contrasted with T cells from primary tumor I. Conversely, T cells from BM displayed a low expression of *MGP* (log2FC = −3.22) and an elevated expression of *PLP1* and *HSPA1A* (log2FC = 2.33 and 2.03, respectively) in comparison with T cells from primary tumor II (Supplementary Table S7).

Furthermore, when comparing T cells from BM with those from primary tumor IV, we observed significant alterations in the expression of genes such as *PLP1* (log2FC = 2.34), *HSPA1A* (log2FC = 2.09), *HBB* (log2FC = −2.93), and *HBA2* (log2FC = −2.44). Moreover, the *TFF3*, *SCGB3A1*, and *PLP1* genes consistently exhibited differential expression in T cells from BM when compared with T cells from primary tumors, suggesting that these genes may be associated with BM (Supplementary Table S7). Subsequent pathway enrichment analysis revealed that dysregulated genes in T cells from BM were associated with crucial lung-cancer-related pathways, such as the PI3K-Akt signaling pathway, ECM-receptor interaction, and MAPK signaling pathway (*p* < 0.05) (Supplementary Table S14).

### 2.6. Stage-Specific Subclustering Unveils Distinctive Profiles of Dendritic Cells in the Tumor Microenvironment

First, cells were clustered into major cell types, as described previously. Subsequently, DC, T cell, and B cell populations were divided into subsets for normalization, dimensionality reduction, and further subclustering stratification analysis. This approach allowed for the detection of heterogeneity within each cell type, considering their inherent diversity.

In a prior investigation, we conducted a detailed subclustering analysis of DCs in BM samples, revealing the presence of CD163+CD14+ DCs [22]. Expanding our focus to primary tumor stages I, II, III, and IV, we investigated the diverse subtypes of DCs, as well as T cells and B cells, in both BM and primary tumors. Our reclassification of DCs into six subsets, including CD1c+ DCs (Langerhans cells, LCs), CD141+ DCs, CD207+CD1a+ LCs, plasmacytoid DCs (pDCs), CD163+CD14+ DCs, and activated DCs, uncovered significant heterogeneity.

**Figure 8:**
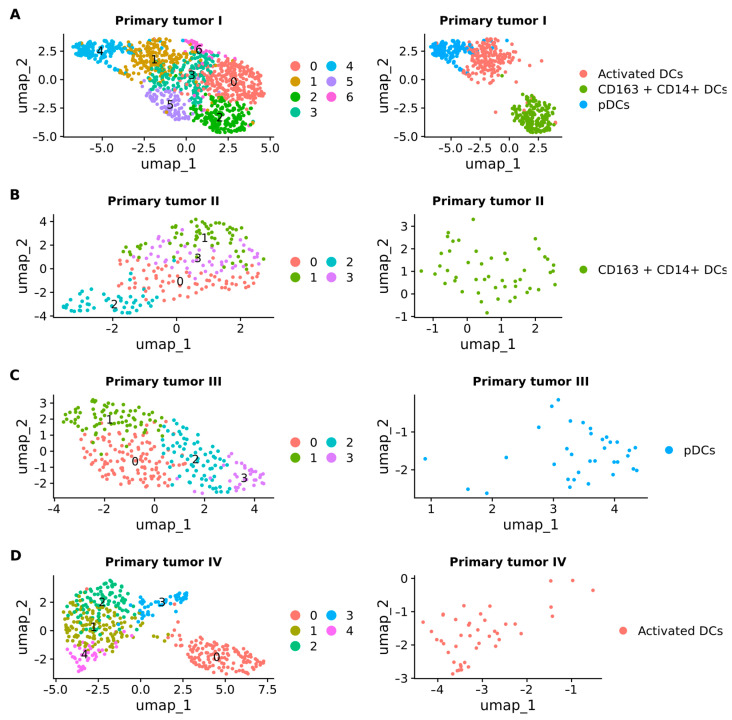
UMAP plots showing the subclusters of dendritic cells (DCs). (**A**) Primary tumor stage I. (**B**) Primary tumor stage II. (**C**) Primary tumor stage III. (**D**) Primary tumor stage IV. pDCs: plasmacytoid DCs.

In primary tumor stage I, we subclustered 1161 DCs into seven distinct subclusters, identifying subcluster 1 as activated DCs (*n* = 211 cells), subcluster 2 as CD163+CD14+ DCs (*n* = 191 cells), and subcluster 4 as pDCs (*n* = 145 cells) (Figure 8A). Moving to primary tumor stage II, we subclustered 232 DCs into four subclusters, with subcluster 3 representing CD163+CD14+ DCs (*n* = 48 cells) (Figure 8B). For primary tumor stage III, our subclustering of 295 DCs into four subclusters revealed cluster 3 as pDCs (*n* = 34 cells) (Figure 8C). Finally, in primary tumor stage IV, we subclustered 457 DCs into five subclusters, pinpointing subcluster 4 as containing activated DCs (*n* = 43) (Figure 8D).

### 2.7. Comprehensive Subclustering of T and B Cells Reveals Stage-Specific Profiles

Afterward, we subclustered T cells obtained from both primary tumors and BM samples into distinct subsets: CD8+ T (naïve, cytotoxic, exhausted), naïve CD4+ T, T regulatory (Treg), T follicular helper, T helper 17, T helper 1, T helper 2, and gamma delta T.

**Figure 9:**
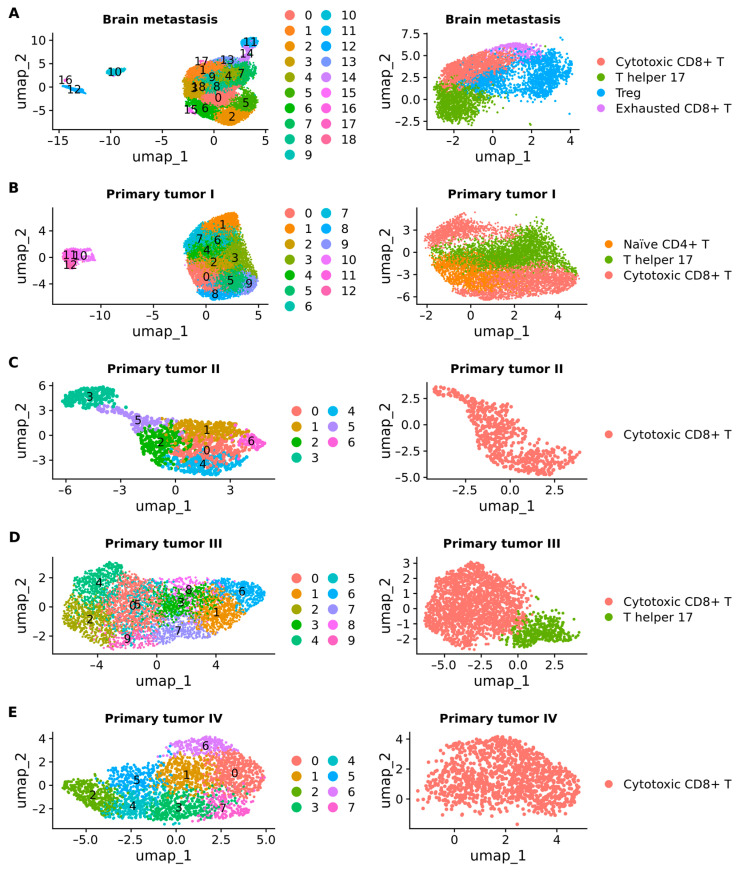
UMAP plots showing the subclusters of T cells. (**A**) Brain metastasis. (**B**) Primary tumor stage I. (**C**) Primary tumor stage II. (**D**) Primary tumor stage III. (**E**) Primary tumor stage IV. Treg: T regulatory.

In BM samples, a total of 13,818 T cells were further subclustered into 19 subclusters. Among these, subcluster 1 demonstrated a cytotoxic profile (*n* = 2670), subcluster 3 represented T helper 17 (*n* = 1257), subclusters 7 and 9 exhibited Treg characteristics (*n* = 1667), and subcluster 13 displayed features of exhaustion (*n* = 315) (Figure 9A). For primary tumor stage I, 13,125 T cells underwent subclustering, resulting in 13 distinct subclusters. Notably, subcluster 0 was identified as naïve CD4+ T (*n* = 1531), subclusters 2 and 7 as T helper 17 (*n* = 2500), and subclusters 3, 5, 8, and 9 as cytotoxic CD8+ T (*n* = 3893) (Figure 9B). Moving to primary tumor stage II, 1373 cells were clustered into 7 subclusters, with clusters 2, 4, and 5 classified as cytotoxic CD8+ T (*n* = 567) (Figure 9C). In primary tumor stage III, 4865 T cells were subclustered into 10 subsets, where subclusters 0, 2, and 4 exhibited cytotoxic features (*n* = 1988), and subcluster 7 represented T helper 17 (*n* = 396) (Figure 9D). Lastly, for primary tumor stage IV, 2696 cells were subclustered into 8 subclusters, with subclusters 0, 1, and 6 identified as CD8+ T cytotoxic (*n* = 1190) (Figure 9E).

**Figure 10:**
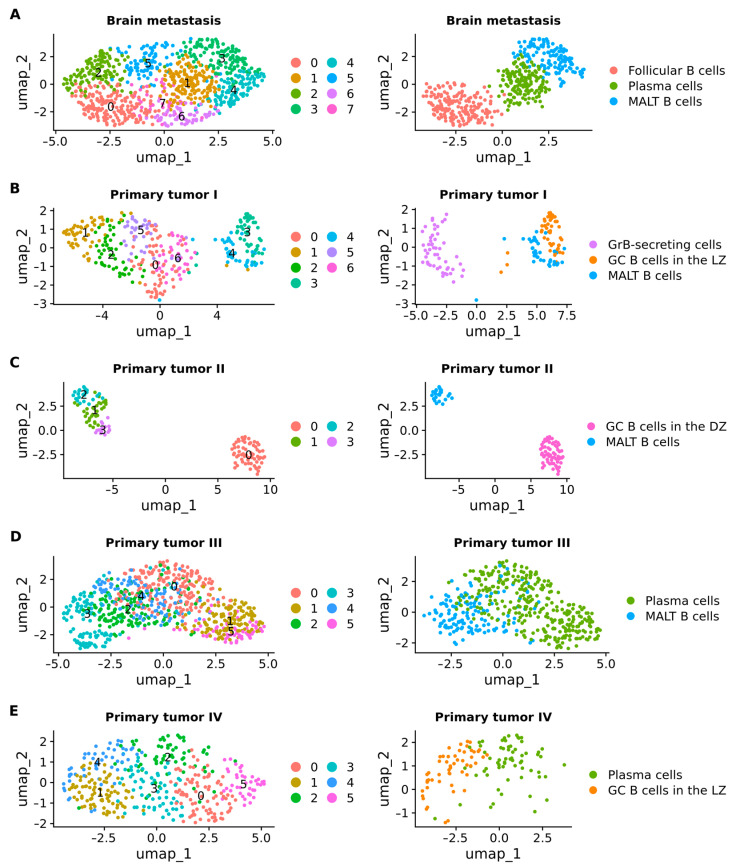
UMAP plots showing the subclusters of B cells. (**A**) Brain metastasis. (**B**) Primary tumor stage I. (**C**) Primary tumor stage II. (**D**) Primary tumor stage III. (**E**) Primary tumor stage IV.

Shifting the focus to B cells, we reclassified them into various subsets, including GC B cells in the dark zone (DZ), GC B cells in the light zone (LZ), GrB-secreting cells, follicular B cells, mucosa-associated lymphoid tissue (MALT) B cells, and plasma cells. In BM samples, a total of 873 B cells were subclustered into 8 distinct subclusters. Notably, subcluster 0 was classified as follicular B cells (*n* = 175), subcluster 1 as plasma cells (*n* = 157), and subcluster 3 as MALT B cells (Figure 10A). In primary tumor stage I, 324 B cells were subclustered into 7 subclusters. Specifically, cluster 2 represented GrB-secreting cells (*n* = 52), subcluster 3 included GC B cells in the LZ (*n* = 46), and subcluster 4 comprised MALT B cells (*n* = 36) (Figure 10B). Transitioning to primary tumor stage II, 133 B cells were subclustered into 5 subclusters, with subcluster 0 defined as GC B cells in the DZ (*n* = 62) and subcluster 2 as MALT B cells (*n* = 23) (Figure 10C). In primary tumors at stage III, 748 cells were subdivided into 6 subclusters, among which subclusters 0 and 1 were identified as plasma cells (*n* = 464), and cluster 2 represented MALT B cells (*n* = 137) (Figure 10D). Finally, 39 B cells from primary tumor stage IV underwent subclustering into 6 subclusters, with subclusters 2 and 4 classified as plasma cells (*n* = 65) and B cells in the LZ (*n* = 52), respectively (Figure 10E).

## 3. Discussion

The TME is a complex ecosystem that surrounds the tumor, composed of a variety of elements including tumor cells, stromal cells, immune cells, extracellular matrix (ECM), blood vessels, chemokines and cytokines, and extracellular vesicles [23,24]. It plays a critical role in tumor development and progression, influencing various stages of tumorigenesis [25,26,27]. Recently, Hanahan revised the hallmarks of cancer and recognized the emerging participation of TME in cancer development [28]. It is well known that the TME is shaped by cancer cells to assist in developing cancer hallmarks, response to stress, stimulation, and treatment, ultimately aiding the survival and migration of tumor cells in an organism [29].

Because of its influence on tumor progression, the TME has received significant attention in the lung cancer literature in recent years, especially in the cancer therapy field [30,31]. Since the TME exerts a key influence on tumor cells and their behavior, the therapeutic approaches for modulating the TME are promising [31]. Some of the strategies that have been explored include the inhibition of macrophage recruitment, the reprogramming of tumor-associated macrophages (TAMs), the depletion of TAMs, and the engineering of TAMs [30,32,33]. Additionally, the effects of other treatment modalities, such as radiotherapy, chemotherapy, anti-EGFR treatment, or photodynamic therapy (PDT), combined with TAM-targeted therapy, have also attracted attention [33]. Furthermore, the targeting of other components of the TME, such as tumor-infiltrating T cells [34], cancer-associated fibroblasts [35], and the ECM [36], has been investigated as a potential therapeutic approach.

While the TME, and therapeutic strategies targeting it, have been extensively researched in lung cancer [17,33,37], the focus has predominantly been on primary tumors, leaving a notable gap in the studies related to BM. Additionally, many earlier clinical trials for immunotherapy in the context of metastatic disease have excluded patients with brain lesions due to poor survival outcomes and concerns regarding the ability of drugs to cross the blood–brain barrier (BBB) [6]. These limitations underscore the urgent need for further investigations into the BM-TME.

The use of scRNA-seq technology has enabled the study of the cellular and molecular heterogeneity of human tumors by distinguishing their different subpopulations [37]. This has proven crucial in understanding the mechanisms of tumor development and progression [38]. With the advancement of single-cell isolation techniques in the TME, high-quality scRNA-seq data, and new computational models for bioinformatics analyses, it has become possible to explore the complexity of the TME in more detail and to study intercellular communication and interactions between tumor cells and non-malignant cells [38,39].

Recent studies using scRNA-seq have explored the BM-TME, leading to valuable insights into its diverse characteristics [19,40,41,42]. Nevertheless, these studies have been limited by small sample sizes, as BM samples are not always surgically removed. Samples of BM are rare, and the samples obtained from surgery are limited compared to other types of samples. This rarity poses a challenge to studying BM. In this study, to address this challenge, we integrated multiple datasets from scRNA-Seq analyses of BM-LUAD. To our knowledge, this is the first comprehensive study of its type.

Our approach allowed us to obtain, after quality control, 79,729 cells from 23 samples of BM and 57,217 from 15 samples of primary tumors. These cells were cataloged into distinct cell types, including immune and non-immune cells. The proportion of each cell type was quantified in both primary tumors and BM, revealing higher proportions of immune cells in both settings. Remarkably, our observations delineated distinct profiles of immunological infiltration into primary tumors and between BM and primary tumors. This aligns with the existing literature indicating immunological differences between primary tumors and metastases in various cancer types, including breast cancer and melanoma [43,44]. For instance, a study found that immune cell infiltration of the primary tumor, not PD-L1 status, is associated with an improved response to checkpoint inhibition in metastatic melanoma [45]. Furthermore, investigations have revealed that metastatic breast cancers are immunologically more inert than the corresponding primary tumors [43]. These dissimilarities in immune infiltration carry substantial implications for therapy responses and patient outcomes [45,46].

Notably, T cells were the dominant immune cell type in primary tumors, while microglia represented the main cell type identified in BM. Similar to our results, in malignant gliomas, microglia are found to be one of the main immune components of a tumor mass [47]. It is well known that microglia serve as the resident macrophages in the brain and are indispensable components of the brain microenvironment, participating in processes of innate immunity and maintaining central nervous system homeostasis [48,49]. In regular homeostatic conditions, microglia remain resting or quiescent, but they become activated in response to disease or injury, including tumor cell invasion [50]. These microglial cells release pro- and anti-inflammatory cytokines that aim to modulate the inflammatory scenario at the site of metastasis [51]. They are also involved in the formation of the pre-metastatic niche in the brain. Furthermore, microglia have been shown to interact with metastatic cancer and immune cells, and their functional plasticity can be modified by these interactions. Such interactions can impact the development of BM, including promoting tumor cell progression and influencing the metastatic colonization process [50,52,53,54,55].

According to our analysis using the ReactomeGSA R package analyse_sc_clusters function to quantify pathways in microglia [56] (Supplementary Figure S1), we found that microglia show a marked upregulation of the COX reaction pathway, well known for its association with the regulation of the inflammatory response [57]. Indeed, other studies have reported that, even in the initial stages, BMs are surrounded by a significant neuroinflammatory response mediated by activated astrocytes and microglia [6,50]. Inflammation is typically referred to as either acute or chronic [58]. Chronic inflammation contributes to cancer development via multiple mechanisms [58].

One potential mechanism involves chronic inflammation generating an immunosuppressive microenvironment, allowing advantages for tumor formation and progression [58]. The immunosuppressive environment in certain chronic inflammatory diseases and solid cancers is characterized by infiltration of immune suppressor cells [58]. In a previous study [22], we demonstrated that BM is highly infiltrated by polymorphonuclear myeloid-derived suppressor cells (PMN-MDSCs), which upregulate the IL-17 signaling pathway. In this previous study, supported by the existing literature, we raised the hypothesis that PMN-MDSCs play an important role in creating an immunosuppressive microenvironment in BM.

It has been demonstrated that MDSCs are a targetable link between chronic inflammation and cancer [58] and contribute to cancer immune evasion by suppressing effector T cell activation, proliferation, trafficking, and viability; inhibiting NKs; and promoting the activation and expansion of Treg cells [58,59,60,61]. Here, the subclustering of T cells (Figure 9) demonstrated that BM was the only group not primarily composed of T cell cytotoxicity, suggesting that MDSCs could be contributing to immune evasion in BM by suppressing effector T cell activation.

As mentioned before, in our previous study, we found PMN-MDSCs enriched in interleukin-17 (IL-17) [22]. IL-17 is a pro-inflammatory cytokine that has been implicated in the recruitment of MDSCs, contributing to immune suppression and tumor progression [62]. The interaction between IL-17 and MDSCs is influenced by the local and systemic levels of interleukin-1 (IL-1), which can promote the accumulation of MDSCs in the tumor microenvironment [63]. IL-17 is produced by T helper 17 (Th17) cells [64]. Th17 are effector cells that promote neuroinflammation [64]. We have previously shown that BM is enriched in the CD163 + CD14+ DC subset, which has a strong Th17 polarizing capacity, as evidenced by the pro-Th17 gene signature [22]. It was demonstrated that the generation of Th17 cells requires certain pro-inflammatory cytokines such as interleukin-23 (IL-23), which are primarily secreted by antigen-presenting cells (APCs) like DCs, macrophages, and B cells [65]. However, in the context of neuroinflammation and neurodegenerative diseases, microglia have also been shown to produce IL-23 [66,67,68,69].

Our data showed that both microglia and DCs upregulated JAK-STAT signaling after interleukin-12 stimulation (Supplementary Figure S2). IL-23 utilizes orthologs of gp130 as part of their receptor complex to signal through the JAK/STAT pathway [70,71]. A study using cell lines with brain-metastatic tropism showed that the polarized phenotype of microglia via JAK2/STAT3 signaling has been implicated in promoting BM from NSCLC by enhancing colonization [54].

Collectively, these results suggest that activated microglia, CD14+ DC, Th17, and PMN-MDSCs are closely interconnected in the context of BM, encompassing chronic inflammation and an immunosuppressive environment. However, there is still a need for further investigation to gain a deeper understanding of the specific roles played by microglia, T cells, and MDSCs in shaping the immunosuppressive BM-TME, as well as their exact contributions to the progression of BM.

By using CellphoneDB to explore intercellular communications through receptor–ligand relationship pairs, we discovered that microglia primarily interact with oligodendrocytes and endothelial cells. One significant interaction was identified between *DLL4* and *NOTCH4*, which demonstrated a relevant association between endothelial cells and microglia and between microglia and oligodendrocytes. The Notch signaling pathway, including DLL4-Notch4 interactions, has been associated with vasculogenic mimicry, tumor recurrence, and prognosis in NSCLC and other malignancies [72,73]. *DLL4* and *NOTCH4* have been investigated for their roles in promoting metastasis and cancer stem cell activities, and their downregulation has been linked to a reduced metastatic burden and the inhibition of cancer stem cells [74]. Therefore, considering both the existing literature and our findings, the *NOTCH4* and *DLL4* genes emerge as potential therapeutic targets for the treatment of BM, and further exploration of their functions in this context is warranted.

Additionally, we found another notable interaction between *VEGFC* and *KDR*, revealing a significant association between microglia and oligodendrocytes (*p* = 0.029). The expression of VEGF and its receptor, KDR, has been correlated with vascularity, metastasis, and proliferation in human colon cancer [75]. Targeting the VEGF pathway, including *VEGF* and *KDR*, has been explored as a potential therapeutic strategy for brain tumors [75].

T cells were found to dominate the immune landscape in primary tumors (Figure 5A). The observed high percentage of T cells among immune-infiltrating cells in NSCLC is in accordance with previous reports [16,22]. We further subclustered these T cells into subtypes, including cytotoxic CD8+ T cells, T helper 17, and naïve CD4+ T cells in primary tumors, and cytotoxic CD8+ T cells, T helper 17, naïve CD4+ T cells, and exhausted CD8+ T cells in BM samples. Cytotoxic CD8+ T cells were the only subtype identified in primary tumors at stages II and IV, and represented the most abundant subtype in stage III.

Tumor-infiltrating T cells, particularly T cytotoxic cells, play an indispensable role in the immune response against tumor cells [76,77]. DCs capture tumor-associated antigens (TAAs) and migrate to lymph nodes, where they present TAAs on major histocompatibility complex (MHC) molecules to naïve T cells, triggering the activation of TAA-specific CD4+ helper T cells or CD8+ cytotoxic T cells, via MHC class II or MHC class I, respectively [78,79,80]. The activation of T cells is highly regulated, requiring the recognition of antigens in the context of appropriate MHC molecules [81]. Several studies have shown that the diversity of the HLA repertoire can directly affect the strength of the anti-tumor immune response [82]. In various types of cancer, including lung cancer, the MHC-I genotype and tumor mutational burden have been found to be predictors of the immunotherapy response [83]. Additionally, the MHC-II signature has been found to be associated with anti-tumor immunity and can predict the anti-PD-L1 therapeutic response of patients with bladder cancer [84].

In this study, we have observed that several genes within the HLA complex are downregulated in BM tissue in comparison to primary tumor stage IV (Figure 7). It is common for cancers to experience HLA downregulation [85]. Tumor cells may escape a T cell attack through HLA downregulation, limiting HLA-dependent immunotherapy to some extent [86,87]. Yang et al. conducted a study that showed how HLA-I downregulation in glioma stem cells was linked with abnormal Wnt/β-catenin activity [88]. In progressing metastases in melanoma patients treated with ipilimumab, HLA class I downregulation was most pronounced in progressing metastases from non-responding patients [89].

Collectively, these results suggest that the downregulation of HLA genes in BM, particularly in the context of lung adenocarcinoma, may serve as an immune evasion mechanism. Our findings illuminate the complexity of the molecular and cellular dynamics of BM-LUAD. Recognizing and understanding such mechanisms is essential for addressing the challenges associated with T-cell-based immunotherapy in the treatment of BM-LUAD.

## 4. Materials and Methods

### 4.1. Data Collection

In this study, we conducted comprehensive analyses by utilizing multiple datasets sourced from the Gene Expression Omnibus (GEO) (available online at: https://www.ncbi.nlm.nih.gov/gds/, accessed on 30 November 2023). The specific datasets used are detailed in Table 1.

### 4.2. Pre-Processing and Quality Control of scRNA-Seq Data

All analyses were carried out in R 4.2.1. The pre-processing and quality control were conducted as previously described [22]. Briefly, Seurat objects were created from individual expression matrices using the “Seurat” R package (version 4.0.2) [90,91]. Cells expressing fewer than 200 or more than 9000 genes, along with those exhibiting a mitochondrial gene percentage exceeding 20%, were excluded. Furthermore, genes expressed in fewer than 3 cells were also excluded. The remaining cells underwent the normalization of gene expression matrices using the NormalizeData function in the Seurat package. To identify the genes that exhibited the highest cell-to-cell variation, Seurat FindVariableFeatures was used to select the top 2000 genes.

### 4.3. Dimensionality Reduction, Clustering, and Cell Type Annotations

The datasets were integrated and the batch effects were removed via canonical correlation analysis and mutual nearest-neighbor anchors using the functions SelectIntegrationFeatures, FindIntegrationAnchors, and IntegrateData from the Seurat package. Following this, we applied data scaling with the Seurat ScaleData function and linear dimensional reduction through principal component analysis (PCA) using the RunPCA function. To visualize both cells and features defining the PCA functions such as DimPlot, VizDimLoadings, and ElbowPlot were employed. The optimal dimensionality of the dataset was determined using the ElbowPlot function. Cell visualization was performed using uniform manifold approximation and projection (UMAP) through the RunUMAP function. We utilized the FindAllMarkers function to identify the differentially expressed genes (DEGs) in each subset or subcluster. The criteria for DEGs were *p*. adj < 0.05 (Wilcoxon rank-sum test) and |log2 FC| > 1. Then, we annotated clusters and subclusters using the CellMarker2.0 database [92] and, after that, we validated the annotation manually using specific cell surface markers previously described in the literature in studies on BM from lung cancer (Supplementary Table S16). Only genes expressed in over 25% of cells with at least a 0.25-fold difference were considered for each cluster and subcluster.

### 4.4. scRNA-Seq Pathway Analysis

To identify the pathways enriched by cell types, we used the analyze_sc_clusters function and extracted the results through the pathways function from the “ReactomeGSA” package (version 1.12.0) [56].

### 4.5. Differential Expression Analysis of Immune Cells and Enrichment Analysis

To identify immune cells’ DEGs between BM and primary tumors, we used the FindMarkers function from the Seurat package. The significance of the difference was determined by using the Wilcoxon rank-sum test with the Bonferroni correction. Genes with |log2 FC| > 1 and an adjusted *p*-value < 0.05 were considered DEGs. The enrichment analysis was conducted as previously described [22]. Briefly, we utilized the enrichGO() function from the R package clusterProfiler (version 4.0.5) [93] to investigate Gene Ontology (GO). To perform the GO enrichment analysis, we obtained the GO annotation file from Gene Ontology (available online at: http://geneontology.org/, accessed on 1 October 2021). For the Kyoto Encyclopedia of Genes and Genomes (KEGG) enrichment analysis, we used the enrichKEGG() function from clusterProfiler. In all analyses, we controlled the false discovery rate (FDR) by adjusting the *p*-value using the Benjamini–Hochberg method. We considered categories with a cutoff of *p*. adj < 0.05 as significant. To visualize the results, we used the Ggplot2 (version 3.4.4) and GOplot (version 1.0.2) packages [94,95].

### 4.6. Cell Communication Analysis

The analysis of cell communication was conducted using CellPhoneDB (version 2.1.7) [96], a publicly available database of receptor–ligand interactions. The cell matrix was normalized using Seurat normalization. The significance of cell communication (*p* < 0.05) and the significant mean were calculated based on the interaction. The results were visualized using the CCPlotR package (version 0.99.3) [97].

## 5. Conclusions

We have presented a detailed overview of the TME of BM-LUAD and primary tumor. By integrating multiple scRNA-seq datasets, we have identified unique immunological infiltration profiles in primary tumors and BM. Our analysis shows that T cells dominate in primary tumors, while microglia are the primary immune cells in BM, emphasizing the importance of the brain microenvironment in shaping the BM-TME.

Our study also highlights the role of chronic inflammation in BM and focuses on PMN-MDSCs and their association with IL-17 signaling. We have discussed the interconnected roles of microglia, T cells, and MDSCs in chronic inflammation within the BM-TME.

We have used CellphoneDB to analyze intercellular communication and have identified significant interactions between microglia, endothelial cells, and oligodendrocytes. This suggests potential therapeutic targets like Notch4 and DLL4. Additionally, the VEGF pathway involving *VEGFC* and *KDR* has shown associations with microglia and oligodendrocytes, providing insights into vascularization and proliferation in BM.

Finally, we identified a downregulation in HLA genes in BM, indicating a potential immune evasion mechanism. Understanding these mechanisms is crucial for addressing challenges associated with T-cell-based immunotherapy in BM-LUAD.

This study provides a crucial basis for researching the molecular mechanisms of, and targeted therapy for, LUAD. The findings emphasize the necessity for continued research to understand the complexities of the BM-TME, which can help pave the way for targeted therapeutic interventions and improved patient outcomes.

## Figures and Tables

**Table 1:** Description of single-cell transcriptomic data used in this study.

Database	Access	Platform	Stage	No. of Samples	No. of Cells *	Ref
Brain metastasis						
GEO	GSE131907	Illumina HiSeq 2500	III/IV	10	29,057	[16]
GEO	GSE202371	Illumina NovaSeq 6000	IV	10	38,476	[16]
GEO	GSE143423	HiSeq X Ten	IV	3	12,196	NA
Primary tumor						
GEO	GSE131907	Illumina HiSeq 2500	I	8	31,025	[16]
GEO	GSE131907	Illumina HiSeq 2500	II	1	3840	[16]
GEO	GSE131907	Illumina HiSeq 2500	III	2	10,282	[16]
GEO	GSE131907	Illumina HiSeq 2500	IV	4	12,070	[16]

* Number of cells after quality control. NA: not available. No.: number.

**Table 2:** Number of cells per tumor stage in primary tumor samples.

	Stage I	Stage II	Stage III	Stage IV
Immune cells				
Macrophages	3801	483	1411	526
NK cells	3439	517	292	732
Neutrophils	651	353		636
Dendritic cells	1161	232	295	457
B cells	324	133	748	390
Mast cells	1480	222	235	
T cells	13,125	1373	4865	2696
Non-immune cells				
Endothelial	456	100	174	153
Fibroblasts			418	228
Epithelial cells	2670	220	467	4970

## Data Availability

The datasets supporting this study’s findings are available in Gene Expression Omnibus (GEO) under accession numbers GSE131907, GSE202371, and GSE143423.

## Supplementary Materials

The following supporting information can be downloaded at: https://www.mdpi.com/article/10.3390/ijms25073779/s1.
