# Supplementary figures and images for: Comprehensive Analysis of Lung Adenocarcinoma and Brain Metastasis through Integrated Single-Cell Transcriptomics
Source: Int J Mol Sci. 2024 Mar 28;25(7):3779. doi: 10.3390/ijms25073779 (PMC11012108; doi:10.3390/ijms25073779)

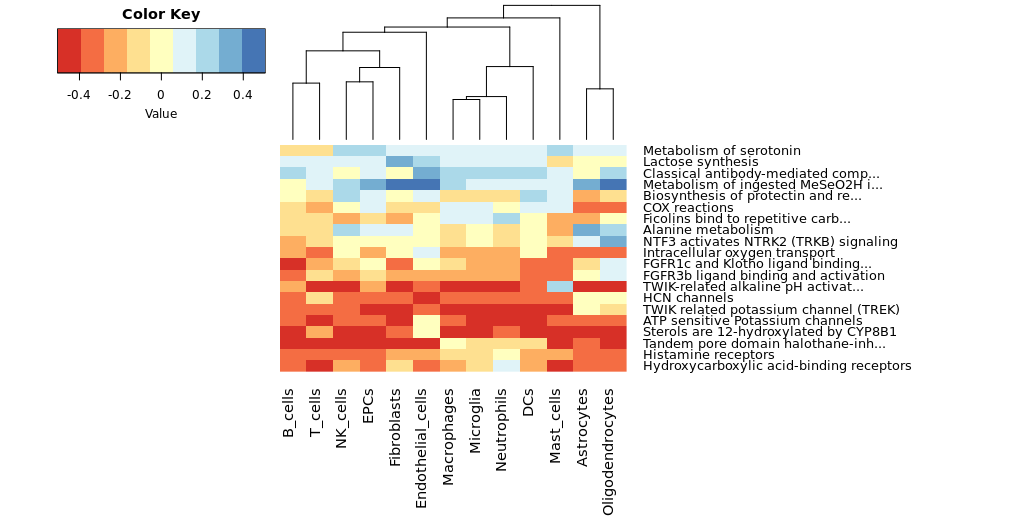

Supplement: Supplementary file 1 [file ijms-25-03779-s001.zip › Supplementary Figure S1.png]

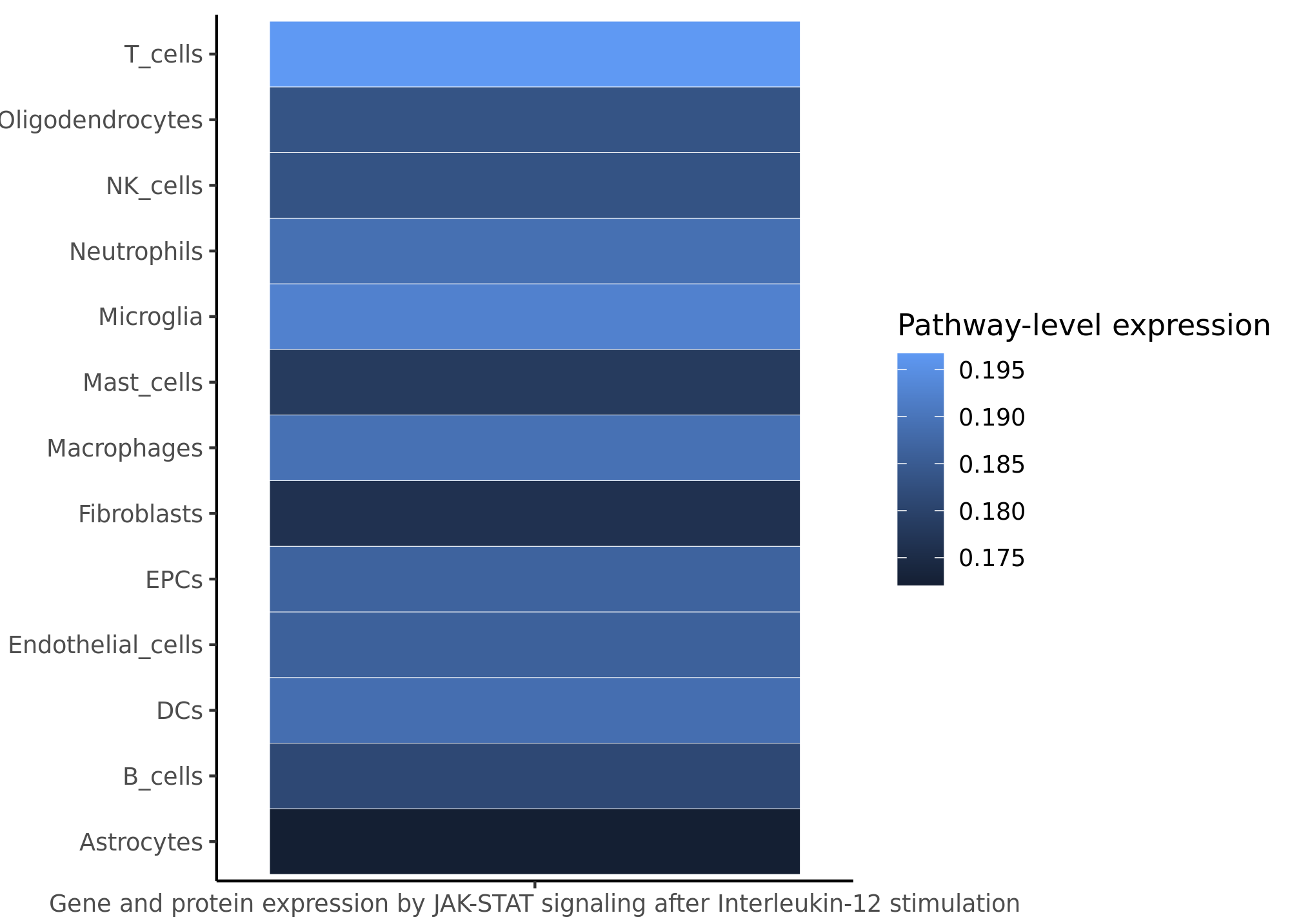

Supplement: Supplementary file 1 [file ijms-25-03779-s001.zip › Supplementary Figure S2.png]

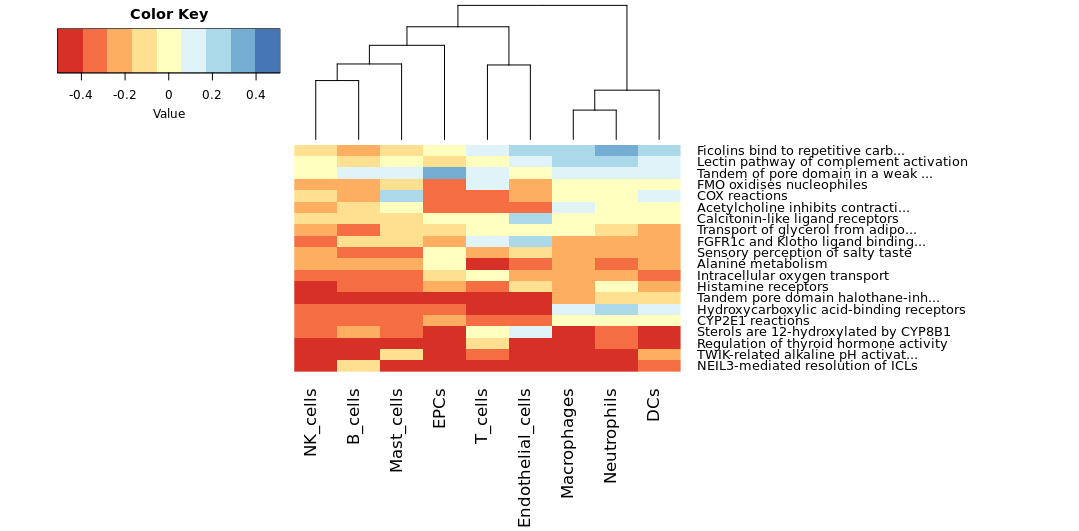

Supplement: Supplementary file 1 [file ijms-25-03779-s001.zip › Supplementary Figure S3.png]

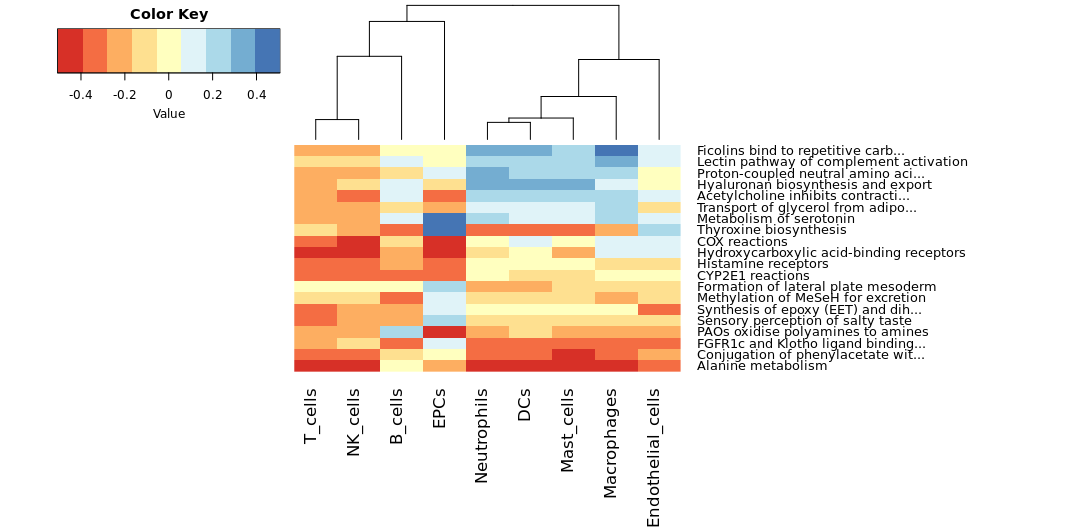

Supplement: Supplementary file 1 [file ijms-25-03779-s001.zip › Supplementary Figure S4.png]

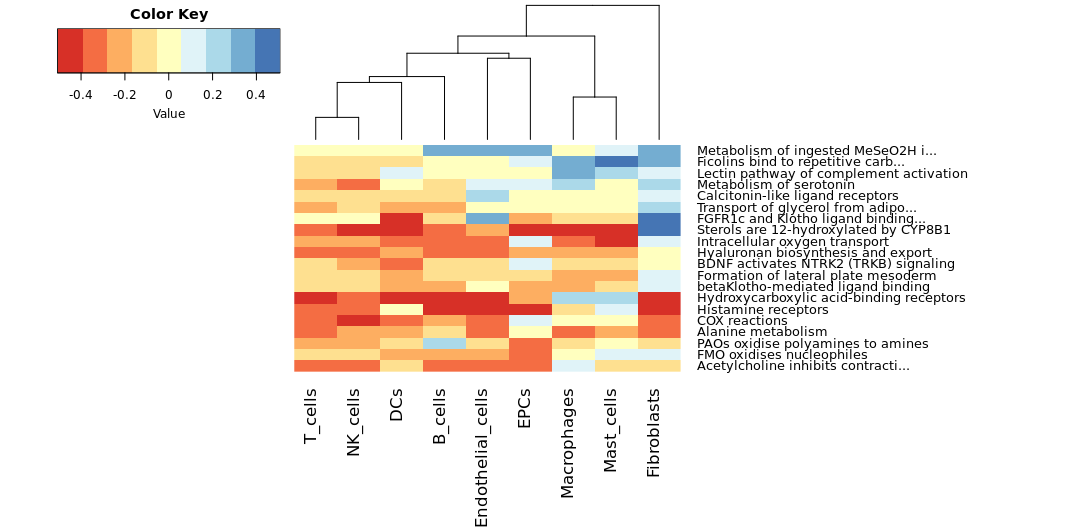

Supplement: Supplementary file 1 [file ijms-25-03779-s001.zip › Supplementary Figure S5.png]

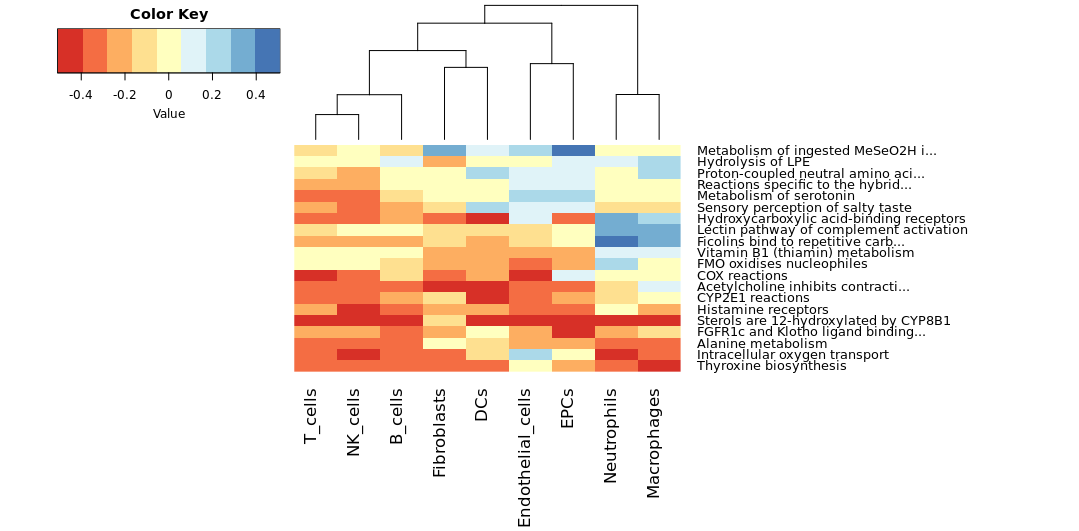

Supplement: Supplementary file 1 [file ijms-25-03779-s001.zip › Supplementary Figure S6.png]
